# Randomised controlled trial on vitreoretinal surgery with and without oral anticoagulants: surgical complications, visual results and perioperative thromboembolic events

**DOI:** 10.1186/s13063-019-3805-6

**Published:** 2019-12-04

**Authors:** Jose Andonegui, Ferran Capdevila, Alicia Zubicoa, Berta Ibáñez

**Affiliations:** 1 Department of Ophthalmology, Complejo Hospitalario de Navarra, 31007 Pamplona, Spain; 2 Instituto de Investigación Sanitaria de Navarra (IdiSNA), Pamplona, Spain; 3 Navarrabiomed, Complejo Hospitalario de Navarra, Universidad Pública de Navarra, Pamplona, Spain; 4 Red de Investigación en Servicios Sanitarios y Enfermedades Crónicas (REDISSEC), Pamplona, Spain

**Keywords:** Pars plana vitrectomy, Anticoagulants, Complications

## Abstract

**Background:**

Vitreoretinal surgery in anticoagulated patients is a challenging situation for vitreoretinal surgeons, who have to choose between being faced with the systemic thromboembolic risks that the interruption of anticoagulation involves, or the intra- and postoperative haemorrhagic risks associated with maintenance of this therapy. So far, no trial has compared, in a prospective and randomized manner, perioperative complications and the visual results associated with continuation or interruption of oral anticoagulant therapy before pars plana vitrectomy (PPV) under retrobulbar anaesthesia. The main objective of this trial is to compare haemostasis-related perioperative complications of PPV in patients maintaining anticoagulant therapy before surgery compared to patients with an interruption in this therapy before surgery.

**Methods:**

Ninety-six patients will be randomly assigned to either the control group, in whom oral anticoagulant therapy will be interrupted and substituted with subcutaneous heparin according to local clinical practice, or the intervention group in whom oral anticoagulant therapy will not be interrupted before surgery. Patients will be stratified according to the oral anticoagulant they were taking (direct or indirect anticoagulation). They will be followed up for 12 weeks, and the primary outcome, and haemorrhagic complications until 15 days after surgery, will be evaluated.

**Discussion:**

This trial will provide novel information on the possibility of continuing anticoagulant therapy during PPV. The benefits expected from the change in the current surgical management paradigm for anticoagulated patients would be a decreased risk in the incidence of perioperative thromboembolic events and the possibility of performing surgery without delay and without the need for patients to change their usual anticoagulation protocol to the more complex and less safe substitutive therapy.

**Trial registration:**

Clinical Trials Register EudraCT, 2018–000753-45. Registered on 11 November 2018.

## Background

The administration of anticoagulant drugs to reduce thromboembolic events is a widespread clinical practice, especially among patients of advanced age [1, 2]. Currently, vitamin K antagonists (acenocumarol) or new direct-acting oral anticoagulants (dabigatran, rivaroxaban, apixaban and edoxaban) are being used. Atrial fibrillation is the most frequent indication, but these drugs are also employed in patients with artificial heart valves, ischaemic cardiopathy, deep venous thrombosis, hypercoagulability states and peripheral vasculopathies. The use of these drugs is constantly expanding, and this fact, together with an ageing population, is expected to produce a notable increase in the number of anticoagulated patients over the coming years.

The indication for pars plana vitrectomy (PPV) is being expanded and is likely to continue in the coming years. PPV in patients undergoing anticoagulation represents a challenge for retinal specialists who must carefully evaluate possible trade-offs between the systemic risks associated with interrupting anticoagulant therapy and the intra- and postoperative risks of performing PPV in these cases. Moreover, interruption of anticoagulant therapy and instauration of substitutive therapy is cumbersome for patients and makes it necessary to postpone surgery for a number of days, a situation that can be inconvenient in urgent PPV indications such as retinal detachment.

Most of the studies performed to date to evaluate the risks of anticoagulant therapy during intraocular surgery have been conducted in patients undergoing cataract surgery [3, 4]. There is currently no consensus with regard to the need or otherwise to interrupt oral anticoagulant therapy before vitreoretinal surgery. Most of the authors that have evaluated this issue have done so in a retrospective manner and agree that continuation of anticoagulant therapy before PPV does not pose a significant risk for these patients [5–10].

So far, no trial has compared the perioperative complications and visual results associated with continuation or interruption of oral anticoagulant therapy before PPV under retrobulbar anaesthesia in a prospective and randomised manner. There is only one study that has evaluated this issue in a prospective but nonrandomised manner, but the authors included a small number of patients, did not standardise the management of anticoagulant agents in the perioperative period and did not record all haemorrhagic complications [11]. Moreover, there is only one study that has evaluated haemorrhagic intraoperative complications associated with PPV in patients under treatment with novel anticoagulant therapy [10].

In order to overcome the shortage of works on this topic, we have designed a randomised clinical trial aimed at comparing haemostasis-related perioperative complications of PPV in patients maintaining anticoagulant therapy before surgery compared to patients who have interruption of this therapy before PPV. Additionally, both the incidence of systemic thromboembolic events after PPV and the changes in visual results after PPV will be compared between the groups.

## Methods and design

A randomised clinical trial with two parallel groups will be conducted at the Ophthalmology Department of the Complejo Hospitalario de Navarra in Spain. Clinical management of the patients will be performed according to local current clinical practice except in the experimental group where oral anticoagulant therapy will not be interrupted before surgery. Patients will be stratified according to the oral anticoagulant they are taking (direct or indirect anticoagulation).

### Primary objective

Our main objective is to compare haemostasis-related perioperative complications of PPV in patients maintaining oral anticoagulant therapy before surgery compared to patients with interruption of this therapy before PPV.

### Secondary objectives

Our secondary objectives are to compare changes in the visual results 3 months after PPV in patients who maintain anticoagulant therapy compared to those with interruption of this therapy before surgery and to compare the incidence of systemic thromboembolic events after PPV in those patients who maintain anticoagulant therapy compared to those with interruption of this therapy before surgery.

### Hypothesis

We hypothesise that anticoagulant therapy maintenance before PPV is not associated with a significant increase in perioperative complications compared to those cases in which this therapy is interrupted before surgery, and that the postoperative visual results are not inferior in those cases in which anticoagulant therapy is maintained compared to those cases in which such therapy is interrupted before surgery.

### Participants, recruitment methods and procedures

**Fig. 1:**
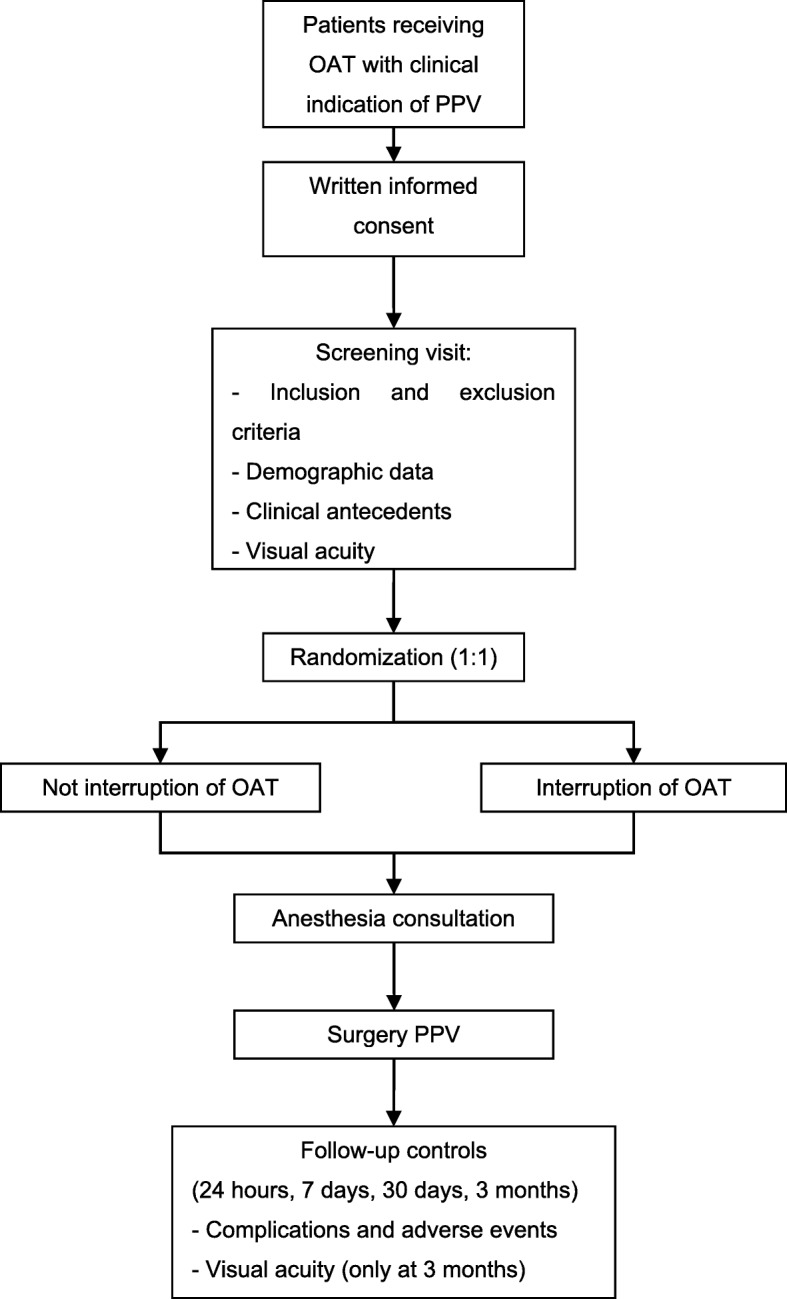
Study timetable and assessments. OAT oral anticoagulant therapy, PPV pars plana vitrectomy

**Fig. 2:**
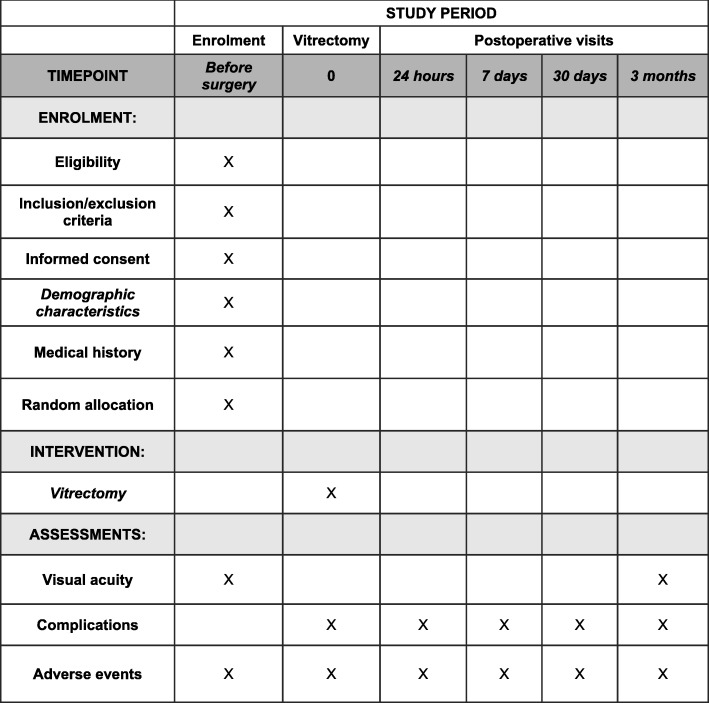
Schedule of enrolment, interventions and assessments

Patients with a possible clinical indication for PPV identified by any ophthalmologist in the reference area are referred to be evaluated by the vitreoretinal surgeons of the Ophthalmology Department in the Complejo Hospitalario de Navarra, who will confirm the surgical indication, in addition to assessing the medical status and checking the current treatments in each case. The surgeon in charge will invite all subjects receiving oral anticoagulant therapy with a clinical indication for PPV to participate in the study. The purposes, methods, objectives and possible risks associated with the study will be suitably explained to them. A patient information sheet and an informed consent form will be subsequently provided. A specific consent form for the surgery will also be provided to participants. The schedule of enrolment, interventions and assessments is summarized in Figs. 1 and 2. A screening visit will be scheduled for patients who decide to participate in the study. In that visit, the inclusion and exclusion criteria will be assessed (Table 1), and patients will be informed of the group to which they will be assigned, together with the date of their operation. A description of the surgical procedure will also be provided during the screening visit. They will also be scheduled to attend an anaesthesia consultation. If the anaesthetist considers that there is a circumstance that contraindicates the interruption of anticoagulant treatment, the patient will be excluded from the trial. After PPV, they will be followed up in order to assess complications, adverse effects and visual acuity for 3 months.

**Table 1:** Inclusion and exclusion criteria

Inclusion criteria	Exclusion criteria
1. Adults over 18 years old of both sexes 2. Clinical indication for PPV alone or associated with facoemulsification or scleral buckling 3. Under treatment with any of the following oral anticoagulants: acenocumarol, dabigatran, rivaroxaban, apixaban or edoxaban 4. Able to give informed consent for PPV and to participate in the trial	1. Patients unable to be operated under retrobulbar anaesthesia 2. Patients on antiplatelet therapy except if the antiaggregation is with acetylsalicylic acid 3. Patients who have recently started oral anticoagulation (less than 1 month before screening) 4. Any medical, psychological, psychiatric, geographic or social situation that could interfere with the participation of the patient in the trial or with the follow-up and adherence with the protocol and the evaluation of the results of the present trial

*PPV* pars plana vitrectomy

### Study randomisation

Upon completion of the screening assessment, participants who satisfy all the inclusion criteria and none of the exclusion criteria will be randomly assigned to the intervention and control groups in a 1:1 allocation ratio. Randomisation will be stratified by oral anticoagulation type: direct anticoagulation, which includes dabigatran, rivaroxaban, apixaban or edoxaban, and indirect anticoagulation, which includes acenocumarol. Randomisation assignment will be computer generated using the ‘randomizr’ library of the R statistical program R 3.4.0, with a complete randomising method based on permutations that guarantees balanced groups. The allocation sequence will be generated by the Methodology Unit of Navarrabiomed. Randomisation will be requested to the Clinical Trials Platform (CTP) of Navarrabiomed by the investigator responsible for patient recruitment. In return, CTP will send to this investigator an answer form by email that includes a randomisation number and the assigned treatment for this patient. Then, the investigator responsible for patient recruitment will give the information about treatment allocation to the anaesthetist, who will inform the patient. Throughout the study, the randomisation will be conducted by the CTP and the randomisation list will be kept by the CTP team for the duration of the study. Randomisation will be conducted without any influence of the investigators. The surgeons in charge, who will measure the outcomes, will be blinded. The data analyst will also be blinded, whereas participants and the rest of the investigators will not be blinded. Unblinding will not be allowed under any circumstance.

### Study arms

In the control group (representing the standard of care), anticoagulant therapy will be interrupted before surgery and patients will receive substitutive treatment with subcutaneous enoxaparin in accordance with the currently employed protocol in the hospital.

To establish substitution therapy, the thrombotic risk of patients is evaluated, taking into account their clinical characteristics. Their renal function is also evaluated by measuring the glomerular filtration rate. In patients undergoing treatment with acenocumarol, if the thrombotic risk is low, they will be treated with enoxaparin 40 mg/24 h; if the thrombotic risk is high and the glomerular filtration rate is less than 30 ml/min, they will be treated with enoxaparin 40 mg/24 h; if the thrombotic risk is high and the glomerular filtration rate is greater than 30 ml/min, they will be treated with enoxaparin 40 mg/12 h. In patients undergoing treatment with the new direct action oral anticoagulants, if the glomerular filtration rate is less than 50 ml/min or if the thrombotic risk is high, the treatment is replaced 5 days before with enoxaparin 40 mg/24 h. If the glomerular filtration rate is greater than 50 ml/min and the thrombotic risk is low, anticoagulant treatment is suspended without the need for substitution treatment.

For the intervention group oral anticoagulant therapy will not be interrupted. Patients will continue their regular oral anticoagulant therapy before and after surgery.

### Pars plana vitrectomy

Patients will undergo PPV with the 23- or 25-gauge technique and under retrobulbar anaesthesia. PPV may be associated with facoemulsification or scleral buckling if there is a clinical indication. Blood pressure will be measured before retrobulbar anaesthesia in all patients. Before surgery, the anaesthetist in charge will verify that the patient has performed the anticoagulant procedure to which he or she was assigned. The International Normalised Ratio will be measured in those patients under treatment with acenocumarol. Retrobulbar anaesthesia will be performed according to the common technique of injecting bupivacaine 0.5% or a mixture of bupivacaine 0.5% and lidocaine 2% with a 25-gauge retrobulbar needle. Epinephrine will not be used. The volume of anaesthetic injected will be 7 ml and the injection may be repeated whenever the surgeon considers it necessary. Apart from retrobulbar anaesthesia, parenteral anxiolytics and analgesics may be administered before or during surgery. Concomitant care or interventions other than usual clinical practice are not expected.

### Primary and secondary endpoints

The primary endpoint is the presence of any of the following complications: 1) retrobulbar or peribulbar haemorrhage after local anaesthesia (preoperative complication); 2) choroidal haemorrhage or incoercible haemorrhage in the anterior chamber or vitreous cavity during surgery (intraoperative complications); and 3) during the first 2 weeks after surgery, anterior chamber haemorrhage, vitreous haemorrhage, intraretinal haemorrhage or choroidal haemorrhage (postoperative complications). These haemorrhagic complications can be divided according to their clinical relevance into major (choroidal haemorrhage and retrobulbar or peribulbar haemorrhage) and minor (anterior chamber haemorrhage, vitreous haemorrhage and intraretinal haemorrhage). The secondary endpoints are: 1) a change in the corrected visual acuity after surgery (measured as the difference between baseline and 3 months after surgery, in the decimal scale and converted into logMAR scale); 2) thromboembolic events during the first 3 months after surgery; and 3) other surgical procedure-related complications during the first 3 months after surgery.

### Safety

All adverse events and serious adverse events which might be related to the study procedures will be recorded, investigated and notified to the regulatory authorities and to the ethics committee by the researchers during the study period, in accordance with Clinical Trials Directive 2001/20/EC.

### Finalisation or interruption of the study

The trial will be considered finished when all the patients have concluded the follow-up period. It may be interrupted under any of the following situations: 1) impossibility of recruiting the number of patients previously determined; 2) noncompliance with the requisites of the protocol; 3) noncompliance with the rules of clinical practice or current legislation; and 4) appearance of unexpected risks that are unacceptable for patients.

### Statistics

#### Sample size

Considering that there is no difference in the probability of not having complications between the standard therapy (anticoagulant interruption) and the experimental therapy (no interruption of anticoagulant), and assuming a 95% confidence level and a 96% probability of not having complications, 48 patients are required in each arm (*n* = 48 × 2) in order to have an 80% probability that the upper limit of the confidence interval excludes a difference of over 10% in favour of the standard group. Calculations are performed by Sealed Envelope Ltd.

#### Data analysis

A description of the sociodemographic and clinical characteristics of the patients involved in this trial will be performed for the complete sample and for each group using a mean with standard deviation or median with interquartile range for the quantitative variables, and using frequencies and percentages for the categorical variables. These characteristics will be compared between the control and experimental groups using parametric tests such as the Student *t* test and Chi-squared test, or nonparametric tests such as the Mann–Witney or Fisher tests. If any of the variables differs significantly between groups, this will be taken into account when comparing the outcomes. The incidence of haemorrhage (pre-, intra- and postoperative) and other categorical secondary variables, such as complications 3 months after surgery, both for the total sample and for each group, will be estimated by means of sample proportion with a 95% confidence interval and from baseline to 3 months after surgery will be compared between groups using parametric or nonparametric tests, such as the Student *t* test and Mann–Whitney test, depending on normality. If any sociodemographic or clinical characteristic differs between groups, lineal and logistic regression models will be used to compare the outcomes between groups, including them as covariates, which will provide adjusted odds ratios for the intervention, together with 95% confidence intervals. Sensitivity analyses will also be performed excluding patients with proliferative vitreoretinopathy or proliferative diabetic retinopathy, patients for whom scleral buckling procedures have been placed, and patients under treatment with acetylsalicylic acid to account for the possible bias induced after having included them. Additionally, a multivariate analysis including age and treatment with acetylsalicylic acid will also be performed to account for possible differences in the distribution of these variables among groups. SPSS software version 21.0 will be used to perform the analysis.

#### Trial governance

The Steering Committee will consist of the Principal Investigator and his research collaborators, which includes clinicians with expertise in ophthalmology. They will be responsible for the recruitment, data collection and completion of case report forms. They will also perform assessments to guarantee adherence to the study protocol. Trial management and data verification will be carried out from the centre’s CTP of Navarrabiomed and data analysis will be conducted by the Unit of Methodology of Navarrabiomed.

#### Regulatory issues

This clinical trial was approved by the local ethics committee (Comité Ético de Investigación con medicamentos de Navarra, Health Department of Navarre Government) and was authorised by the Spanish Agency of Medicines and Medical Devices. This study will be performed in accordance with the standard of Good Clinical Practice and with the current European and Spanish laws that are applicable. The study will be performed in accordance with ethical principles that have their origin in the Declaration of Helsinki and are consistent with International Council for Harmonisation of Technical Requirements for Registration of Pharmaceuticals for Human Use/Good Clinical Practice, and applicable regulatory requirements (Additional file 1).

## Discussion

Vitreoretinal surgery in anticoagulated patients is a complex situation in which retinal surgeons have to choose between being faced with the systemic thromboembolic risks that the interruption of anticoagulation involves, or the intra- and postoperative haemorrhagic risks associated with maintenance of this therapy.

The interruption of anticoagulant therapy before surgery is not innocuous. The risk of thromboembolic events is increased after this treatment is interrupted and during the interval after its re-instauration, when the International Normalised Ratio is below therapeutic levels. The risk associated with the interruption of anticoagulant therapy oscillates between 0.02% in low-risk patients with atrial fibrillation and 0.42% in high-risk patients with artificial heart valves [12]. Cases of serious complications, and even death, have been described in patients who had interrupted anticoagulant treatment before surgery [13–15]. Some investigators have also suggested that anticoagulant therapy interruption could produce a rebound hypercoagulability state due to the alteration of the balance between vitamin K-dependent factors and proteins C and S [16–19]. Moreover, it is a situation that makes it necessary to postpone surgery for a number of days, and this can compromise visual recovery in some surgical indications such as retinal detachment.

There are only a few studies that have evaluated the possibility of anticoagulant therapy continuation before PPV, and most of the authors agree that this circumstance does not involve a particular risk for patients [5–10]. In this respect, Brown and Mahmoud did not find either intraoperative or postoperative complications attributable to the anticoagulation in 27 eyes undergoing diabetic PPV [5]. Chandra et al. did not find an increase in perioperative or postoperative complications of PPV, in particular haemorrhage, in 60 patients taking warfarin [6]. Fu et al. had good visual results in 25 patients undergoing PPV while on warfarin therapy, with only one highly myopic patient suffering a submacular haemorrhage, which resolved spontaneously with a good final visual acuity [7]. Mason et al. describe one transient vitreous haemorrhage (1.6%) among 64 PPVs performed on patients anticoagulated with warfarin, without significant differences from a control group of 110 individuals [8].

Even though some authors have found an increased risk of haemorrhagic complications in anticoagulated patients undergoing PPV, the incidence has been low, without statistical significance, and without serious consequences for visual acuity [9–11, 20, 21]. Dayani and Grand analysed 54 patients who underwent PPV while taking warfarin and found four patients who suffered postoperative vitreous haemorrhage, all of which resolved spontaneously requiring no further surgery [9]. Grand and Walia studied 36 eyes of 33 patients who underwent PPV while being treated with novel anticoagulant therapy and reported that four eyes (11.1%) experienced postoperative vitreous haemorrhage after which two eyes (5.5%) required repeat surgical intervention and two eyes (5.5%) cleared spontaneously [10]. Meillon et al. [11] and Oh et al. [20] report 6.6% and 7% of haemorrhagic complications, respectively, after PPV in patients undergoing anticoagulation therapy, in both studies without visual consequences in the long term. Narendran and Williamson report one case of postoperative choroidal haemorrhage, but only 7 of the 541 patients in their study were taking warfarin during PPV [21].

In contrast to other eye surgeries, vitreoretinal surgery has been considered to pose a high haemorrhagic risk [22]. This means that any perioperative bleeding could have devastating consequences. However, according to the current scientific evidence, this statement cannot be considered to be strictly true. The minor haemorrhagic complications (anterior chamber haemorrhage, vitreous haemorrhage and intraretinal haemorrhage) that can be expected after PPV have a minimal clinical relevance and spontaneous resolution without long-term consequences is usually the norm, with the systemic risk from anticoagulant therapy interruption being much greater. The major haemorrhagic complications are very rare. Most choroidal haemorrhages resolve spontaneously, although some cases with massive haemorrhage and retinal apposition may be difficult to manage. With regard to retrobulbar haemorrhages, they also resolve spontaneously, although their appearance may cause the surgical intervention to be delayed. This circumstance has led to many authors proposing a change in this paradigm and considering PPV as having moderate or even low haemorrhagic risk and considering continuing anticoagulant therapy before surgery [23–25], an assertion that the present trial will attempt to assess.

The main strength of this trial and the most relevant difference compared to previous studies on the topic is its prospective and randomised design. The most important limitations are the small number of patients to be recruited and the low expected incidence of complications, two circumstances that could make it difficult to achieve results of statistical significance.

In conclusion, with this trial we will attempt to provide novel information assessing the possibility of anticoagulant therapy continuation during PPV. The benefits expected from the change in the current surgical management paradigm of anticoagulated patients would be a decreased risk in the incidence of perioperative thromboembolic events and the possibility of performing surgery without delay and without the need for patients to change their usual anticoagulation protocol to the more complex and less secure substitutive therapy.

### Trial status

The study is currently in the process of recruiting participants. Recruitment of participants commenced on 1 April 2019 and will be completed in April 2021. This article is based on protocol version 1.0, 5 October 2018.

## Supplementary information

**Additional file 1.** SPIRIT 2013 checklist: recommended items to address in a clinical trial protocol and related documents.

## Data Availability

The complete study protocol is available on request. The datasets generated and/or analysed during the current study are available from the corresponding author on reasonable request.

## Abbreviations

CTP: Clinical Trials Platform
PPV: Pars plana vitrectomy
